# Supplementary material for: Short term effect of antimicrobial photodynamic therapy and probiotic L. salivarius WB21 on halitosis: A controlled and randomized clinical trial
Source: PLoS One. 2024 Jul 2;19(7):e0297351. doi: 10.1371/journal.pone.0297351 (PMC11218947; doi:10.1371/journal.pone.0297351)
Supplement: S2 File — Protocol published in English. (DOCX) [file pone.0297351.s003.docx]

**UNIVERSITY NINTH OF JULY**

**GRADUATE PROGRAM IN BIOPHOTONICS APPLIED TO HEALTH SCIENCES**

**Pamella de Barros Motta**

**COMPARATIVE STUDY BETWEEN PHOTODYNAMIC THERAPY WITH ANNAMOR AND LED AND PROBIOTICS IN HALITOSIS REDUCTION - RANDOMIZED CONTROLLED CLINICAL TRIAL**

**Sao Paulo-SP**

**2019**

**ABSTRACT**

Halitosis is a term that defines any odor or bad smell originating from the oral cavity, which may have local or systemic origin. This project aims to verify whether the treatment with antimicrobial photodynamic therapy (aPDT) and the treatment with the use of probiotics are effective against it. Fifty-two UNINOVE students or employees, aged between 18 and 25 years old, with a diagnosis of halitosis, showing sulfhydride (SH2) ≥ 112 ppb on gas chromatography will be selected. Participants will be randomly divided into 4 groups of 13, which will receive different treatments: Group 1: treatment with brushing, dental floss and tongue scraper; Group 2: brushing, flossing and aPDT applied to the dorsum and middle third of the tongue; Group 3: floss brushing and probiotics; Group 4: brushing, flossing, aPDT and probiotics. The results of halimetry will be compared before, immediately after treatment, seven days after and thirty days after treatment. A microbiological analysis of the tongue coating will be carried out at these same times. Quantitative analysis will be performed using real-time PCR. Data normality will be checked using the Shapiro-Wilk test, and, in the case of normality, the Analysis of Variance (ANOVA) test will be applied, and, in the case of non-parametric data, the Kruskal-Wallis test will be used. To analyze the results of each treatment in the two study periods, the Wilcoxon test will be used.

**Descriptors:** Halitosis, Photodynamic Therapy, Bixa orellana, Probiotics

**Clinical Trials:** NCT03996044

**1. INTRODUCTION**

Halitosis is a term that defines any odor or bad smell from the oral cavity, which may have a local or systemic origin [1].

Volatile Sulfur Compounds (CVS) are chemical components that are related to halitosis, they are: hydrogen sulfide (H2S), methylmercaptans (CH3SH) and dimethylsulfide (CH3SCH3) [2-5].

There are different methods for diagnosing halitosis: clinical evaluation, known as an organoleptic test, a subjective method that consists of feeling the smell exhaled through the mouth and nose, and then quantifying this odor using a scale. CVS can be measured using sulfide monitors. And gas chromatography is the most appropriate method for diagnosing halitosis of any origin, as it measures the 3 main sulfide gases, methylmercaptan and dimethylsulfide [6-8]. Despite having a complex etiology, anaerobic bacteria are identified as the main cause [6]. The prevalence of halitosis is high, and it is possible to find values above 50% in the literature [9].

Conventional treatments used to control halitosis basically consist of the use of dentifrices and mouthwashes containing bactericidal substances, use of a lingual scraper, treatment of caries lesions and periodontal disease, in addition to controlling xerostomia [10]. Some studies suggest that amine fluoride has a positive effect in reducing halitosis [9].

Studies show that alternative treatments such as Antimicrobial Photodynamic Therapy (aPDT) [12, 13, 14] and probiotics have been used in an attempt to control halitosis [9, 10, 15].

aPDT is a treatment in which a photosensitizing agent is used that, in the presence of light, produces oxygen free radicals leading to cell death [16].

Probiotics are defined as microorganisms that provide beneficial effects to the health of the host when absorbed by it. They are often used in food and fermented products, in addition to being used in pharmaceutical manipulations [17].

Advantages of alternative approaches are reduced tissue damage and avoidance of bacterial resistance.

**2. JUSTIFICATION**

Halitosis is considered an important social factor, as it interferes with interpersonal relationships. In addition to generating concerns related to the physical health of the individual, it can cause psychological changes, leading to a social barrier [18].

The realization of the project is justified due to the scarcity of studies that evaluate the reduction of halitosis through photodynamic therapy and the use of probiotics, presenting annatto as a photosensitizer and LED as a more accessible light source for dentists. Although methylene blue combined with red laser has already been used for this purpose, this study will evaluate the effect of aPDT with annatto and LED to reduce halitosis. The fact that annatto is red facilitates the work combined with a light emitting diode (LED) and allows its use on a larger scale. In addition, the LED is low cost and most dentists already have the device in their offices. Protocols with the use of LED to reduce halitosis have already been developed and obtained positive results, observing recolonization after 7 days of treatment [12, 19]. The purpose of this project is to continue these protocols, adding the use of probiotics and analysis by gas chromatography up to 30 days after treatment to assess its effectiveness.

The use of probiotics in dentistry presents an innovative treatment, capable of modifying the oral microbiota, especially with regard to halitosis, since the oral microbiota is complex and a major challenge in the development of protocols for the prevention and treatment of the disease [20] .

The present study proposes a controlled clinical trial to compare the effect of photodynamic therapy and the use of probiotics in controlling halitosis.

**3. HYPOTHESIS**

*Experimental hypothesis:* There is a decrease in halitosis after the use of photodynamic therapy using blue dye and red LED. There is a decrease in halitosis after treatment with probiotics. There is microbiological change after antimicrobial photodynamic therapy. There is microbiological change after treatment with probiotics.

*Null hypothesis:* There is no change in halitosis after the use of photodynamic therapy employing the use of blue dye and red LED. There is no change in halitosis after treatment with probiotics There is no microbiological change after antimicrobial photodynamic therapy. There is no microbiological change after treatment with probiotics.

**4. OBJECTIVE**

The objective of the present study is to verify if the treatment with aPDT, using annatto as a photosensitizer and the LED as a light source, is effective in the immediate reduction of halitosis when evaluated by gas chromatography, as well as to compare this method with the use of a tongue scraper, most commonly used conventional method, use of dental floss and brushing with amine fluoride toothpaste (Elmex®) and use of probiotics.

Perform a quantitative microbiological analysis of the bacteria present on the tongue coating before and after treatment, using real-time PCR.

**5. MATERIAL AND METHODS**

An invitation will be made and participants who are interested in participating will be selected. 52 students or employees of UNINOVE with a diagnosis of halitosis, showing sulfhydride (SH2) ≥ 112 ppb in the gas chromatography will be included. Participants will be divided through block randomization into four groups (n=13), according to the treatment to be performed (Figure 1). Group 1: treatment with brushing, dental floss and tongue scraper; Group 2: brushing, flossing and aPDT applied to the dorsum and middle third of the tongue; Group 3: floss brushing and probiotics; Group 4: brushing, flossing, aPDT and probiotics. The results of halimetry will be compared before, immediately after treatment, seven days after and thirty days after treatment. A microbiological analysis of the tongue coating will be carried out at these same times. Quantitative analysis will be performed using real-time PCR. This work will be sent to the Research Ethics Committee of UNINOVE and because it is a randomized clinical trial and seeking greater transparency and quality of this research, we will use the recommendations of CONSORT (Consolidated Standards of Reporting Trials)

**5.1. Inclusion criteria**

Participants of both genders, aged 18 to 25 years, with a diagnosis of halitosis showing sulfhydride (SH2) ≥ 112 ppb in gas chromatography will be included in this research.

**5.2. Exclusion Criteria**

Individuals with dentofacial anomalies (such as cleft lip, palatine and nasopalatine fissures), undergoing orthodontic and/or orthopedic treatment, undergoing oncological treatment, with systemic alterations (gastrointestinal, renal, hepatic), under antibiotic treatment up to 1 day will be excluded from the study. month before the survey and pregnant women.

**Randomization**

Randomization will be performed using the randomizer.org website and the order will be placed in a brown envelope and the participant will remove the envelope at the time of treatment.

**Procedures**

Because it is a randomized clinical trial and seeking greater transparency and quality of this research, we will use the CONSORT recommendations (Consolidated Standards of Reporting Trials) (figure 1).

**Individuals from the UNINOVE dental clinic**

**Excluded**

**Recruitment**

**Halimetry**

**Excluded SH2<112ppb**

**SH2 diagnosis>112ppb**

**n=52**

**Group4**

**n=13**

**Group 3**

**n=13**

**Group 2**

**n=13**

**Group 1**

**n=13**

**Brushing, flossing and aPDT e probiotics**

**Brushing, flossing and probiotics**

**Brushing, flossing and scraper**

**Brushing, flossing and aPDT**

**New halimetry immediately after treatment**

**Halimetry after 7 days**

**Halimetry after 30 days**

**Analysis of results**

**Figure 1:** Flowchart of activities.

**6. INTERVENTIONS**

**Group 1 - Brushing, flossing and scraper**

1- Initial measurement

2- Collection of lingual coating with swab

3- Tongue scraping

4- Oral Hygiene Guidelines

5- Halimetry immediately after scraping

6- Halimetry after 7 and 30 days

**Group 2 – Brushing, flossing and aPDT**

1- Initial measurement

2- Collection of lingual coating with swab

3- Application of annatto spray on the back of the tongue for 2 minutes

4- Application of LED in 6 points

5- Oral Hygiene Guidelines

6- Halimetry immediately after aPDT

7- Halimetry after 7 and 30 days

**Group 3 – Floss brushing and use of probiotics**

1- Initial measurement

2- Collection of lingual coating with swab

3- Delivery and guidance on the use of probiotics

4- Oral Hygiene Guidelines

5- Halimetry on the 14th day of treatment with probiotics

6- Halimetry 7 and 30 days after completion of treatment

**Group 4- Brushing, flossing, aPDT and probiotics**

1- Initial measurement

2- Collection of lingual coating with swab

3- Application of annatto spray on the back of the tongue for 2 minutes

4- Application of LED in 6 points

5- Delivery and guidance on the use of probiotics

6- Oral Hygiene Guidelines

7- Halimetry on the 14th day of treatment with probiotics

8- Halimetry 7 and 30 days after completion of treatment

**Sample calculation**

To calculate the sample size, data from the work by Costa da Mota et al. (Effect of photodynamic therapy for the treatment of halitosis in adolescents - a controlled, microbiological, clinical trial).

Initially, an error was established $err=\left| \bar{x_{1}}-\bar{x_{2}} \right|$, onde$\bar{x_{1}}$ e $\bar{x_{2}}$ are the mean values of the baseline groups to periodontal treatment with PDT. From this error, the effect size was calculated, given by $\frac{err}{\sqrt{\sigma_{1}^{2}+\sigma_{2}^{2}}}$ where $\sigma_{1}^{2}$e $\sigma_{2}^{2}$ are the variances of groups one and two, respectively.

Assuming that the studied groups have normal or approximately normal distribution, that the sample size will be large enough and that a two-tailed test will be used, for a significance level α = 0.05 and maintaining the power of the test 1-β = 0.90, we have an n=13 for each group.

Figure 3 shows that with a total sample size of 39 subjects, that is, three groups with 13 samples each, the statistical difference must be demonstrated by keeping the test power greater than or equal to 0.90. If the hypothesis of normality of distributions is rejected, the sample size should be corrected by approximately 5%.

Figure 2: Test power adjustment as a function of the total sample size.

In figure 2 it is observed that with a total sample size of 52 subjects, that is, four groups with 13 samples each, that the statistical difference must be demonstrated keeping the power of the test greater than or equal to 0.90. If the hypothesis of normality of distributions is rejected, the sample size should be corrected by approximately 5%.

*Halimetry*

The collection of oral air will follow the manufacturer's guidelines (Oral ChromaTM Manual Instruction), where the participant will be instructed to rinse their mouth with cysteine (10 mM) for 1 minute, then remain with their mouth closed for another 1 minute. A syringe from the same manufacturer designed to collect mouth air will be introduced into the participant's mouth. For 1 minute, the participant will remain with his mouth closed, breathing through his nose, without touching the syringe with his tongue. The plunger will be pulled out, we will again empty the syringe into the participant's mouth and again we will pull the plunger to fill the syringe with the breath sample. We will clean the tip of the syringe with gauze to remove moisture from the saliva, place the gas injection needle on the syringe, and adjust the plunger to 0.5 ml. The collected gases are injected into the inlet port of the device with a single movement (Figure 4).

**Figure 4:** Process of carrying out the halimetry.

From the analysis of the VSCs captured by the system, we have:

- Sulfidride: origin mainly from the bacteria present on the back of the tongue. Values above 112 ppb are indicators of halitosis.

- Methylmercaptan: predominantly higher in periodontal pockets. Values up to 26 ppb are considered normal. Periodontal disease typically results in a high methylmercaptan/sulfhydride ratio (>3:1)

- Dimethylsulfide: it can either be of periodontal or systemic origin (intestinal, hepatic, pulmonary). It can also be temporarily caused by eating certain foods and drinks. It is possible to distinguish between dimethylsulfide of oral origin and that of systemic origin, by comparing the results of halimetry with and without the cysteine challenge (cysteine 10 mM, i.e., 16 mg of cysteine in 100 ml of water distilled – 16 mg%). The perception threshold for dimethylsulfide is the lowest, 8 ppb. Other odors (not VSCs) may appear in a peak prior to the theoretically first peak, which is the sulfhydride.

To avoid changes in halimetry, participants will be instructed to follow the following guidelines: 48 hours before the evaluation, avoid eating foods with garlic, onions and strong spices, alcohol consumption and use of mouthwash. On the day of the evaluation, in the morning, they can eat up to a maximum of 2 hours before the exam, abstain from coffee, candies, chewing gum, oral and personal hygiene products with perfume (aftershave, deodorant, perfume, creams and/or tonic) and brushing will only be done with water.

*Microbiological analysis*

The lingual coating samples will be collected using 1 sterile swab that will be passed on the surface of the dorsum of the tongue with a back and forth movement 10 times. The samples will be deposited in sterile tubes that will be identified and stored at -80 C until they are analyzed. After thawing, the samples will be submitted to the vortex for 1 minute. For bacterial DNA extraction, the samples will be submitted to a boiling bath for 10 minutes and then centrifuged at 10,000 rpm for 10 minutes. The supernatant will be placed in a new microtube containing 100μL of phenol/chloroform/isoamyl alcohol (25:24:1), followed by ethanol precipitation. Purified DNA will be resuspended in TE buffer. The levels of P. gingivalis, T. forsythia and T. denticola will be analyzed by quantitative PCR. Quantitative analysis will be performed by real-time PCR using a Step One Plus Real-Time PCR System thermal cycler (Applied Biosystem, Foster City, CA, USA) and the products detected by fluorescence using the Quantimix Easy SYG Kit (Biotools, Madrid, Spain), following the protocol recommended by the manufacturer. For the reaction will be used 10 µl of SYBR Green, 0.5 µl template DNA, 200 mM of each primer (P.gingivalis CATAGATATCACGAGGAACTCCGA TT and AAACTGTTAGCAACTACCGATGTGG; T.forsythia GGGTGAGTAACGCGTATGTAACCT and ACCCATCCGCAACCAATAAA; T. denticola CGTTCCTGGGCCTTGTACA and TAGCG ACTTCAGGTACCCTCG; Universal for bacteria CCATGAAGTCGGAATCGCTAG and GCTTGACGGGCGGTGT) in a total volume of 20 µl. For the standard curve, reactions containing as template DNA 2 to 2X105 copies of the analyzed gene (16S rRNA) will be performed using pTOPO plasmids in which the 16S genes of 14 different organisms will be cloned. As a negative control, sterile milliQ water will be added instead of template DNA. Reactions for 16S rRNA will be performed with initial denaturation at 95°C for 2 minutes, followed by 36 cycles of 94°C for 30 seconds, 55°C for 1 minute and 72°C for 2 minutes and final extension at 72°C for 10 minutes 46. Fluorescence will be detected after each cycle and graphed using Step One Plus Real-Time PCR System software (Applied Biosystem, Foster City, CA, USA). To ensure the specificity of products detected by fluorescence and to avoid detection of primer dimers, detection will be performed one degree below the dissociation temperature of the amplicons. All samples will be analyzed in duplicate and each plasmid dilution for the standard curve in triplicate. The purpose of the microbiological evaluation will be to verify the effectiveness of photodynamic therapy for the treatment of halitosis, complementing the clinical evaluation.

*Antimicrobial Photodynamic Therapy (aPDT)*

An LED light curing device – Valo Cordless Ultradent® will be used, an office device with coupled radiometer, spectrum of 440-480nm and irradiance of 450mW/cm. At the time of performing the aPDT, only the participant to be treated and the responsible professional will be present, both wearing specific glasses for eye protection. The active LED tip will be coated with disposable transparent plastic (PVC) (avoiding cross-contamination and for hygiene reasons) and the professional will be properly dressed.

1 aPDT session will be performed with the photosensitizer (FS) annatto manipulated at a concentration of 20% (Formula e Ação®) in spray, to be applied in sufficient quantity to cover the middle third and dorsum of the tongue (5 sprays) for 2 minutes to incubation. The excess will be removed with a sucker in order to keep the surface moist with the FS itself, without using water. Six points will be irradiated with a distance of 1 cm between the points, considering the halo of light scattering and effectiveness of aPDT. The apparatus will be previously calibrated with a wavelength of 395-480 nm, for 20 seconds per point, energy of 9.6J, and the light will be irradiated so that a halo of 2 cm in diameter per point is formed. Table 1 contains all parameters used.

Table 1: LED parameters.

| Wave-length (nm) | 395-480 |
| --- | --- |
| Operating mode | Contínuo |
| Average radiant power (mW) | 480 |
| Polarization | aleatória |
| opening diameter (cm) | 0.9 |
| Irradiance at aperture (mW/cm2) | 762 |
| Beam profile | Top hat |
| irradiated area (cm2) | 3.14 |
| target irradiance (mW/cm2) | 153 |
| Exposition time (s) | 20 |
| Fluency (J/cm2) | 6.37 |
| radiant energy (J) | 9.6 |
| Number of irradiated points | 6 |
| total irradiated area (cm2) | 18.8 |
| Number of sessions | 1 |
| total radiant energy (J) | 57.6 |

*Tongue scraping*

The lingual scraping will be performed by the same operator in all participants. Posterior-anterior movements will be performed with the scraper on the lingual dorsum, followed by cleaning the scraper with gauze. This procedure will be performed ten times in each participant, with the aim of standardizing the mechanical removal of the tongue coating.

*Treatment with probiotics*

Pharmaceutical compounded capsules containing strains of Lactobacillus salivarius WB21 (6.7 x 108 CFU) and xylitol (280mg) will be used. 42 capsules will be delivered to each participant, who must ingest 1 capsule, 3 times a day after meals, for 14 days.

*Brushing with amine fluoride toothpaste*

All 52 participants will be instructed to brush with toothpaste containing amine fluoride in its composition (Elmex®) and use dental floss 3 times a day after meals for 30 days.

*Statistical analysis*

Data from the Oral ChormaTM will be analyzed for normality using the Shapiro-Wilk test. If the hypothesis of normality is accepted, the analysis of variance (ANOVA) will be used followed by the Tukey test when necessary. To analyze the treatment results in the two periods of the study, the T test for paired data will be used. If the hypothesis of normality is rejected, the Kruskal-Wallis test will be used followed by the Student-Newman-Keuls test, when necessary. To analyze the results of each treatment in the two study periods, the Wilcoxon test will be used.

**4- DISCUSSION**

Halitosis is considered an important social factor, as it interferes with interpersonal relationships. In addition to generating concerns related to the physical health of the individual, it can cause psychological changes, leading to a social barrier [18].

There is a scarcity of studies that evaluate the reduction of halitosis through photodynamic therapy and the use of probiotics, presenting annatto as a photosensitizer and LED as a more accessible light source for dentists. Although methylene blue combined with red laser has already been used for this purpose, this study will evaluate the effect of aPDT with annatto and LED to reduce halitosis. The fact that annatto is red facilitates the work combined with a light emitting diode (LED) and allows its use on a larger scale. In addition, the LED is low cost and most dentists already have the device in their offices.

The use of probiotics in dentistry presents an innovative treatment, capable of modifying the oral microbiota, especially with regard to halitosis, since the oral microbiota is complex and a major challenge in the development of protocols for the prevention and treatment of the disease [19] .

The present study proposes a controlled clinical trial to compare the effect of photodynamic therapy and the use of probiotics in controlling halitosis.

| ***Mês/Ano →***  ***Atividades*** | 03/19 | 04/19 | 05/19 | 06/19 | 07/19 | 08/19 | 09/19 | 10/19 | 11/19 | 12/19 | 01/20 | 02/20 | 03/20 | 04/20 | 05/20 | 06/20 | 07/20 | 08/20 | 09/20 | 10/20 | 11/20 | 12/20 | 01/21 | 02/21 |
| --- | --- | --- | --- | --- | --- | --- | --- | --- | --- | --- | --- | --- | --- | --- | --- | --- | --- | --- | --- | --- | --- | --- | --- | --- |
| ***Encaminhar para Comitê de Ética*** |  |  |  |  | X |  |  |  |  |  |  |  |  |  |  |  |  |  |  |  |  |  |  |  |
| ***Revisão***  ***da Literatura*** | X | X | X |  |  |  |  |  |  |  |  |  |  |  |  |  |  |  |  |  |  |  |  |  |
| ***Escrever Material***  ***Método*** |  |  |  | X | X | X | X | X |  |  |  |  |  |  |  |  |  |  |  |  |  |  |  |  |
| ***Triagem dos participantes*** |  |  |  |  |  |  |  |  | X | X | X | X | X | X |  |  |  |  |  |  |  |  |  |  |
| ***Execução da Metodologia*** |  |  |  |  |  |  |  |  |  |  |  |  |  |  | X |  |  |  |  |  |  |  |  |  |
| ***Análise dos Dados*** |  |  |  |  |  |  |  |  |  |  |  |  |  |  |  | X | X |  |  |  |  |  |  |  |
| ***Escrever Resultados*** |  |  |  |  |  |  |  |  |  |  |  |  |  |  |  |  |  | X | X | X |  |  |  |  |
| ***Escrever Discussão e Conclusão*** |  |  |  |  |  |  |  |  |  |  |  |  |  |  |  |  |  |  |  |  | X | X | X |  |
| ***Encaminhar Artigo para Publicação*** |  |  |  |  |  |  |  |  |  |  |  |  |  |  |  |  |  |  |  |  |  |  |  | X |

**SCHEDULE**

**REFERENCES**

1. ARMSTRONG, Brenda L.; SENSAT, Michelle L.; STOLTENBERG, Jill L. Halitosis: a review of current literature. **American Dental Hygienists' Association**, v. 84, n. 2, p. 65-74, 2010.
2. CALIL, CM.; MARCONDES, FK. Influence of anxiety on the production of oral volatile sulfur compounds. Life Science, v. 79, n. 7, p. 660–4, 10 jul. 2006.
3. SPRINGFIELD, J. et al. Spontaneous fluctuations in the concentrations of oral sulfurcontaining gases. J Dental Res, v. 80, n. 5, p. 1441–1444, 2001.
4. TANGERMAN, A; WINKEL, E. G. The portable gas chromatograph OralchromaTM: a method of choice to detect oral and extra-oral halitosis. J Breath Res, v. 2, n. 1, mar. 2008.
5. TOLENTINO, E. D. S.; CHINELLATO, L. E. M.; TARZIA, O. Saliva and tongue coating pH before and after use of mouthwashes and relationship with parameters of halitosis. J Appl Oral Sci, v. 19, n. 2, p. 90–4, abr. 2011.
6. PORTER, S. R.; SCULLY, C. Oral malodour (halitosis). Bmj, v. 333, n. 7569, p. 632-635, 2006.
7. KARA, C. et al. Effect of Nd: YAG laser irradiation on the treatment of oral malodour associated with chronic periodontitis. Int Dent J, v. 58, p. 151–158, 2008.
8. KARA, C; TEZEL, A; ORBAK, R. Effect of oral hygiene instruction and scaling on oral malodour in a population of Turkish children with gingival inflammation. Int J Paediatr Dent, v. 16, n. 6, p. 399–404, nov. 2006.
9. BICAK, Damla Aksit. A current approach to halitosis and oral malodor-A mini review. The open dentistry journal, v. 12, p. 322, 2018.
10. SUZUKI, Nao et al. Induction and Inhibition of Oral Malodor. Molecular Oral Microbiology, 2019.
11. SCULLY, C.; GREENMAN, J. Halitology (breath odour: aetiopathogenesis and management). Oral diseases, v. 18, n. 4, p. 333-345, 2012.
12. DA CIARCIA, Ana Carolina Costa et al. Action of antimicrobial photodynamic therapy with red leds in microorganisms related to halitose. Medicine, v. 98, n. 1, 2019.
13. KELLESARIAN, Sergio Varela et al. Effect of antimicrobial photodynamic therapy and laser alone as adjunct to mechanical debridement in the management of halitosis: A systematic review. Quintessence International, v. 48, n. 7, 2017.
14. COSTA DA MOTA, Ana Carolina et al. Effect of photodynamic therapy for the treatment of halitosis in adolescents–a controlled, microbiological, clinical trial. Journal of biophotonics, v. 9, n. 11-12, p. 1337-1343, 2016.
15. YOO, Jun-Il et al. The Effect of Probiotics on Halitosis: a Systematic Review and Meta-analysis. Probiotics and antimicrobial proteins, v. 11, n. 1, p. 150-157, 2019.
16. HOPE, Chris K.; WILSON, M. Induction of lethal photosensitization in biofilms using a confocal scanning laser as the excitation source. Journal of Antimicrobial Chemotherapy, v. 57, n. 6, p. 1227-1230, 2006.
17. SALMINEN, Seppo et al. Demonstration of safety of probiotics—a review. International journal of food microbiology, v. 44, n. 1-2, p. 93-106, 1998.
18. ELIAS, Marina Sá; FERRIANI, Maria das Graças Carvalho. Aspectos históricos e sociais da halitose. Revista Latino-Americana de Enfermagem, v. 14, n. 5, 2006.
19. GONÇALVES, Marcela Leticia Leal et al. Photodynamic therapy with Bixa orellana extract and LED for the reduction of halitosis: study protocol for a randomized, microbiological and clinical trial. Trials, v. 19, n. 1, p. 590, 2018.
20. MAHASNEH, Sari; MAHASNEH, Adel. Probiotics: a promising role in dental health. Dentistry journal, v. 5, n. 4, p. 26, 2017.

**Term of Free and Informed Consent for Participation in Clinical Trial:**

**Participant's name:______________________________________________________ Address:_____________________________________________________ Contact phone number:____________________City:________________ZIP code:________**

**Email:________________________________________________________**

**1. Title of the Experimental Work: COMPARATIVE STUDY BETWEEN PHOTODYNAMIC THERAPY WITH ANNAMOR AND LED AND PROBIOTICS IN HALITOSIS REDUCTION - RANDOMIZED CONTROLLED CLINICAL TRIAL**

**2.Objective:** To evaluate whether treatment with LED (a light) and a dye (photodynamic therapy, and use of probiotics are effective in the treatment of bad breath.

**3.Justification:** The justification for this research is the search for an alternative to conventional treatment, producing longer-lasting results and more comfort during treatment.

**4. Procedures of the Experimental Phase:** You are being invited to receive treatment for bad breath, using the LED (a light) with a dye to eliminate bacteria or taking probiotics (which are food products that contain live microorganisms whose ingestion brings health benefits), or the conventional method (tongue scraping), at the Uninove clinic.

You will carry out an evaluation of the presence or absence of bad breath and you will be able to participate in one of the four groups of this research according to the draw.

Group 1 will undergo a lingual scraping and receive guidance on brushing and flossing. Group 2 will receive a treatment with a dye spray and a blue light, as well as guidance on brushing and flossing. Group 3 will receive treatment with probiotics and guidance on brushing and flossing. And group 4 will receive treatment with a dye spray and blue light and also probiotics, in addition to brushing and flossing guidance.

Before and after treatment, an evaluation of bad breath and tongue scraping will be performed. Bad breath will be evaluated again after 7 days and 3 days.

**5.Discomfort or Expected Risks:** Participants may feel self-conscious about bad breath. Participants receiving dye treatment may experience tongue sensitivity. The probiotic presents no risk or side effect because they present microorganisms that are already present in the body.

**6. Measures to protect against risks:** The Team will be available if the participant is sensitive to the immediate removal of the product. To avoid embarrassment, the evaluation and treatment will be carried out in a private room with only the participant and the researcher present.

**7. Research Benefits:** Receive treatment for bad breath.

**8. Existing Alternative Methods:** None.

**9. Withdrawal of Consent:** the participant is free to withdraw his consent at any time and stop participating in the study, without prejudice. It is important to point out that in case you withdraw from participating in the study, you will not have any prejudice in relation to your academic or professional activities at the university.

**10. Guarantee of Confidentiality:** The researchers ensure the privacy of the participants regarding the confidential data involved in the research.

**11. Forms of Reimbursement for Expenses arising from Participation in the Research:** There will be no reimbursement.

**12. Place of Research:** The research will be carried out at Uninove Dental Clinic, located at Rua Vergueiro, 235/249 – 2nd basement - Liberdade, São Paulo - SP, 01504-001, Telephone: (11) 2633-9000.

**13.** Research Ethics Committee (CEP) is an interdisciplinary and independent collegiate, which must exist in institutions that carry out research involving human beings in Brazil, created to defend the interests of research participants in their integrity and dignity and to contribute to the development of research within ethical standards (Norms and Regulatory Guidelines for Research Involving Human Beings – Res. CNS nº 466/12 and Res. CNS 510/2016). The Ethics Committee is responsible for evaluating and monitoring the research protocols in terms of ethical aspects. Uninove Ethics Committee address: Rua. Vergueiro nº 235/249 – 12th floor - Liberdade – São Paulo – SP CEP. 01504-001 Phone: 3385-9010 comitedeetica@uninove.br. Opening hours of the Ethics Committee: Monday to Friday – From 11:30 am to 1:00 pm and from 3:30 pm to 7:00 pm

**14.** Full name and telephone numbers of Contact Researchers: Prof. Dr Sandra Kalil Bussadori (011) 98381-7453, and student Pamella de Barros Motta- (011) 97397-6458.

**15.** Eventual intercurrences that may arise during the research may be discussed by the proper means.

Sao Paulo, 20__.

**16.** Post-Information Consent:

I, ________________________________________________, after reading and understanding this information and consent form, understand that my participation is voluntary, and that I can leave the study at any time, without prejudice. I confirm that I have received a copy of this consent form, and I authorize the carrying out of the research work and the dissemination of data obtained only in this study in the scientific community.

____________________________________________________

Participant Signature

(All pages must be initialed by the research participant)

**17.** I, ________________________________________ (Researcher responsible for this research), certify that:

a) Considering that ethics in research implies respect for human dignity and the protection due to participants in scientific research involving human beings;

b) This study has scientific merit and the team of professionals duly mentioned in this term is trained, capable and competent to perform the procedures described in this term;

____________________________________

Responsible Researcher Signature
