# Supplementary material for: Short term effect of antimicrobial photodynamic therapy and probiotic L. salivarius WB21 on halitosis: A controlled and randomized clinical trial
Source: PLoS One. 2024 Jul 2;19(7):e0297351. doi: 10.1371/journal.pone.0297351 (PMC11218947; doi:10.1371/journal.pone.0297351)
Supplement: Attachment — Submitted filename: Response to Reviewers.docx [file pone.0297351.s006.docx]

Response to Reviewers

Reviewer #1: The authors have well addressed all issues raised by the reviewer. No further adjustments are needed.

Answer: We would like to thank the reviewer once again.

Reviewer #2: Major Revisions:
1- Abstract: Please revise the following statement to improve clarity. "The clinical data showed no normality, they were treated using the Kruskal-Wallis test ..." Consider the following. "The clinical data failed to follow a normal distribution; therefore, comparisons were made using the Kruskal-Wallis test..."

Answer: Firstly, we would like to thank the reviewer for revising our manuscript once again. The suggested correction was made and highlighted in the text.

2- Abstract and Lines 350-352: If the microbiological data was normally distributed, why was a non-parametric test, the Kruskal-Wallis rank sum test used? An analogous parametric test such as an ANOVA would be more powerful if the distribution of the data is normal.

Answer: Actually, data distribution was not normal. However, we do agree with the reviewer, the writing was not clearly expressing that. Therefore, we have changed and highlighted the sentence in the text, as follows: “For the microbiological data, seeing as the data failed to follow a normal distribution, the Kruskal-Wallis rank sum test was performed with Dunn's post-test.”

3- Abstract, Results: Provide a p-value to support the primary result.

Answer: p-values have been added and highlighted in the Abstract.

4- The authors should consider providing tests of interaction effects, specifically time by group interaction effects rather than repeatedly applying Kruskal-Wallis tests.

Answer: Other tests have been used in our paper, such as Friedman ANOVA (for dependent measures) followed by appropriate posthoc tests, when necessary (Dunn’s test for independent measurements and pairwise Wilcoxon signed-rank test with Ryan-Holm stepdown Bonferroni procedure for adjustment). In the microbiological results analysis, the Kruskal-Wallis rank sum test was also followed by the Dunn's post-test. The application of these tests has been mentioned both in the Results and in the Abstract of the manuscript.

Minor Revisions:
1- The first paragraph under the "Results" section should be renamed "Statistical analyses".

Answer: The correction has been made and highlighted in the text.
