# Supplementary material for: Short term effect of antimicrobial photodynamic therapy and probiotic L. salivarius WB21 on halitosis: A controlled and randomized clinical trial
Source: PLoS One. 2024 Jul 2;19(7):e0297351. doi: 10.1371/journal.pone.0297351 (PMC11218947; doi:10.1371/journal.pone.0297351)
Supplement: Attachment — Submitted filename: Response to Reviewers.docx [file pone.0297351.s007.docx]

Response to Reviewers

Reviewer #1: In the present study, the authors have well addressed all issues raised by the reviewer. The manuscript can be accepted.

Answer: We would like to thank the reviewer once again.

Reviewer #2: Major revisions:
Line 325: Instead of performing between group comparisons at each time point individually. Use a model that allows for testing the interaction of time by treatment effect. A comprehensive reanalysis is needed.
Process for testing interaction effects:
If the interaction effect is significant, provide an interpretation of the results, but do not test main effects because the tests for main effects are uninteresting in light of significant interactions. If interaction effects are non-significant, drop the interaction effects from the model and test the main effects. Determining which results to present when testing interactions is often a multi-step process.

Answer: We sincerely appreciate your insightful feedback on the statistical analysis approach employed in our study. Your comments have provided valuable insights into alternative methodologies, and we carefully consider your recommendations.

It is acknowledged that the Two-way repeated measures ANOVA test is well-suited for research designs similar to ours but that present an approximately parametrically distributed data. However, given the non-parametric nature of our dataset, applying this method directly may not be appropriate, as it assumes different characteristics inherent in non-parametric data. Other alternative approaches, such as Mixed Effects Models or Multilevel Models also rely on the assumption of normality of residuals. Unfortunately, this assumption cannot be guaranteed by our data.

In light of the non-parametric nature of our data, we opted for the use of the Friedman Test and Kruskal-Wallis Test. These methods are specifically designed to accommodate the non-normal distribution and ranking structure inherent in non-parametric data. To maintain the overall significance level at 0.05, we recognize the importance of implementing corrections for local p-values, such as the Bonferroni or the Ryan-Holm stepdown Bonferroni procedure, even though they may impact test power. Additionally, any interaction within or between groups would have been detected by such tests and would also be observed in the descriptive statistics.

Considering the specific outcomes of our analysis, we observed high p-values (> 0.60) in the between-groups analysis, consistent with our clinical observations. Even with a different statistical approach, these p-values would likely remain above 0.05, thereby preserving the study's conclusions. Similarly, the "Before" and "After" analysis yielded very low p-values (p < 0.001), and alternative statistical approaches are unlikely to alter these results significantly.

We also acknowledge that the chosen statistical models are well-recognized and understood by our target audience. Introducing a more uncommon model might add complexity without commensurate benefits in terms of the level of certainty and overall information provided in the work.

Should you have specific suggestions for a statistical model that could enhance the readability and comprehension of our work while aligning with the assumptions of our data, we would be more than willing to discuss and explore those possibilities.

Minor revision:
1- Line 316: Consider replacing "treated" with "compared".

Answer: The change has been made and highlighted in the text, as requested.

We would like to thank the reviewer, for revising our paper once again.
